# Tuberculosis treatment outcomes among people living with HIV diagnosed using Xpert MTB/RIF versus sputum-smear microscopy in Botswana: a stepped-wedge cluster randomised trial

**DOI:** 10.1186/s12879-019-4697-5

**Published:** 2019-12-16

**Authors:** Tefera Agizew, Violet Chihota, Sambayawo Nyirenda, Zegabriel Tedla, Andrew F. Auld, Unami Mathebula, Anikie Mathoma, Rosanna Boyd, Anand Date, Sherri L. Pals, Phenyo Lekone, Alyssa Finlay

**Affiliations:** 1 Centers for Disease Control and Prevention, Gaborone, Botswana; 2 Faculty of Health Sciences, Department of Public Health, University of the Witwatersrand, Johannesburg, South Africa; 3 Faculty of Medicine, University of Botswana, Gaborone, Botswana; 4 Aurum Institute, Johannesburg, South Africa; 5 Division of Global HIV and Tuberculosis, Centers for Disease Control and Prevention, Atlanta, GA USA; 6 Division of Tuberculosis Elimination, Centers for Disease Control and Prevention, Atlanta, GA USA

**Keywords:** Tuberculosis, Treatment, Outcome, Xpert MTB/RIF, Smear

## Abstract

**Background:**

Xpert® MTB/RIF (Xpert) has high sensitivity for diagnosing tuberculosis (TB) compared to sputum-smear microscopy (smear) and can reduce time-to-diagnosis, time-to-treatment and potentially unfavorable patient-level treatment outcome.

**Methods:**

People living with HIV (PLHIV) initiating antiretroviral therapy at 22 HIV clinics were enrolled and underwent systematic screening for TB (August 2012–November 2014). GeneXpert instruments were deployed following a stepped-wedge design at 13 centers from October 2012–June 2013. Treatment outcomes classified as an unfavorable outcome (died, treatment failure or loss-to-follow-up) or favorable outcome (cured and treatment completed). To determine outcome, smear was performed at month 5 or 6. Empiric treatment was defined as initiating treatment without/before receiving TB-positive results. Adjusting for intra-facility correlation, we compared patient-level treatment outcomes between patients screened using smear (smear arm)- and Xpert-based algorithms (Xpert arm).

**Results:**

Among 6041 patients enrolled (smear arm, 1816; Xpert arm, 4225), 256 (199 per 2985 and 57 per 1582 person-years of follow-up in Xpert and smear arms, respectively; adjusted incidence rate ratio, 9.07; 95% confidence interval [CI]: 4.70–17.48; *p* < 0.001) received TB diagnosis and were treated. TB treatment outcomes were available for 203 patients (79.3%; Xpert, 157; smear, 46). Unfavorable outcomes were reported for 21.7% (10/46) in the smear and 13.4% (21/157) in Xpert arm (adjusted hazard ratio, 1.40; 95% CI: 0.75–2.26; *p* = 0.268). Compared to smear, in Xpert arm median days from sputum collection to TB treatment was 6 days (interquartile range [IQR] 2–17 versus 22 days [IQR] 3–51), *p* = 0.005; patients with available sputum test result had microbiologically confirmed TB in 59.0% (102/173) versus 41.9% (18/43), adjusted Odds Ratio [aOR], 2.00, 95% CI: 1.01–3.96, *p* = 0.048). In smear arm empiric treatment was 68.4% (39/57) versus 48.7% (97/199), aOR, 2.28, 95% CI: 1.24–4.20, *p* = 0.011), compared to Xpert arm.

**Conclusions:**

TB treatment outcomes were similar between the smear and Xpert arms. However, compared to the smear arm, more patients in the Xpert arm received a TB diagnosis, had a microbiologically confirmed TB, and had a shorter time-to-treatment, and had a lower empiric treatment. Further research is recommended to identify potential gaps in the Botswana health system and similar settings.

**Trial registration:**

ClinicalTrials.gov Identifier: NCT02538952. Retrospectively registered on 2 September 2015.

## Background

The usefulness of Xpert® MTB/RIF, a rapid molecular diagnostic test, for intensified tuberculosis (TB) case finding has been well demonstrated in several clinical studies [1, 2]. The HIV infection rate in Botswana is one of the highest in the world with adult HIV prevalence of about 19% [3], and TB is a leading cause of mortality, as high as 40%, in this population, like other sub-Saharan settings [4, 5]. Following the World Health Organization (WHO) recommendation in December 2010, the Botswana Ministry of Health and Wellness adopted Xpert MTB/RIF into the national TB diagnostic algorithm [6]. Xpert MTB/RIF was implemented in phases via the Xpert MTB/RIF Package Rollout Evaluation Study (XPRES).

Xpert MTB/RIF has a high sensitivity for diagnosing TB among people living with HIV (PLHIV) compared to sputum-smear microscopy (smear), and can potentially reduce time-to-diagnosis and time-to-treatment leading to improved TB treatment outcomes [7, 8]. On the basis of its accuracy, shorter turnaround time, and potentially reduced loss-to-follow-up, Xpert MTB/RIF may results in earlier diagnosis and anti-TB treatment initiation to improve patient-level clinical outcomes [7, 9, 10]. Previous studies conducted in South Africa and Brazil, comparing Xpert MTB/RIF and smear, showed no difference in patient-level outcomes [8, 10, 11]. More research on the impact of Xpert MTB/RIF on patient-level treatment outcomes in programmatic settings is needed. Factors associated with clinical outcomes, such as time-to-diagnosis, time-to-treatment initiation and empiric treatment (treatment without or before receiving positive test results) [12] among patients diagnosed with TB via Xpert MTB/RIF or smear need further investigation.

Within the context of XPRES in HIV care and treatment settings in Botswana, we investigated whether Xpert MTB/RIF reduces unfavorable outcomes (treatment failure, death, and loss-to-follow-up), reduces time-to-diagnosis and time-to-treatment, and reduces the use of empiric treatment among patients treated for drug-sensitive TB compared to smear.

## Methods

### Study design and populations

We conducted a multi-center, cluster randomised trial (CRT) called the Xpert Package Rollout Evaluation Study (XPRES) using a Stepped-wedge design trial (Trial registration: ClinicalTrials.gov Identifier:NCT02538952, retrospectively registered on 2 September 2015, available on the website, https://clinicaltrials.gov/ct2/show/NCT02538952). This study is a sub-anlayis of the main XPRES study. See the protocol for full study details, including study populations, sample size, and study procedures [13]. A stepped-wedge rather than parallel group design was chosen because the Xpert package was expected to be beneficial for patients and the trial was part of a national rollout.

A cluster was defined as an HIV care and treatment clinic. Twenty-two clusters, located at five district hospitals and 17 primary healthcare facilities, were purposively selected to: (1) be representative of HIV treatment clinics in Botswana, and (2) have new Antiretroviral Therapy (ART) initiation rates sufficient to meet sample size requirements per protocol. At these 22 clusters, individual patients were eligible for study enrollment if they were new HIV clinic attendees and not prisoners at the time of the first HIV clinic visit from August 2012–November 2014. All eligible patients were enrolled in two consecutive phases: (1) a prospective phase where smear based TB diagnostic algorithm was used (smear arm), and (2) a prospective phase whereby Xpert MTB/RIF based TB diagnostic algorithm was used (Xpert arm).

XPRES enrolment began in 2012 as part of Botswana’s national Xpert MTB/RIF rollout, together with intensified TB case finding activities and strengthening HIV patient retention interventions at 22 HIV treatment clinics before phased implementation of 13 GeneXpert instruments, nine at a peripheral laboratory and four at point of care peripheral clinics. GeneXpert installation occurred over 9 months in a stepped-wedge design, and patients enrolled before the Xpert intervention were tested using smear and post-intervention tested by Xpert. Because of the nature of the study design (a stepped-wedge) and the sensitivity difference assumed between smear and Xpert testing, the sample proportion was just over 1:2.

### Sample size

This is sub-study of main trial (XPRES) and as described previously [13], to answer the first XPRES primary study question with > 80% power and alpha at 0.05, assuming that smear and Xpert TB diagnostic algorithm sensitivities are about 62.5% [14] and 82.4% [2], ,respectively, about 9614 new HIV clinic enrollees needed to be enrolled (3266 smear arm and 6348 Xpert arm, after GeneXpert instrument roll-out). In summary, with funding restrictions, XPRES ended up enrolling 6041 (1816 smear and 4225 Xpert arm). However, as described in the published protocol, the > 80% power to detect smear versus Xpert TB diagnostic sensitivities was maintained when the assumptions were adjusted to the interim culture-positive TB prevalence rate together with smear sensitivity estimated at < 52.5% and Xpert sensitivity > 82.5% [13].

### .Inclusion criteria for prospective cohorts

- All consenting adult patients (we use the legal definition of adult: > 18 years old) newly registered in the prospective period.
- All persons newly registered in the prospective period and aged 7–17 who assent to enrollment and for whom the guardian provides consent for enrollment.
- All children < 7 years old newly registered in the prospective period and for whom consent for enrollment has been provided by the guardian.

**Exclusion criteria for prospective cohorts**:

- Patient (or patient’s guardian if patient is < 18 years old) does not provide consent.
- Patients aged 7–17 years old who do not provide assent.
- Patient (or patient’s guardian if patient is < 18 years old) declines to provide contact information for themselves.
- Patient (or patient’s guardian if patient is < 18 years old) declines to allow study staff to contact them by phone and in person if they miss a study visit.
- All prisoners.

### Randomisation procedure

The selected 22 clusters received TB diagnostic services from 13 testing centers (nine laboratories and four point of care). The randomization we used in our methodology was not at individual patient level, rather for the order of placement of the 13 GeneXpert instrument. In our cluster 4 GeneXpert instrument served 9 HIV clinics as point of care and another 13 HIV clinics were served by 9 GeneXpert placed at centralized laboratory.

After obtaining ethical approvals and agreement to participate in the study from Ministry of Health and Wellness at a central level and health management team at the selected study facilities, the study statistician randomly selected one of the roll-out permutations. Over the 9 months (from October 2012–June 2013) all the 13 GeneXpert instrument sites were activated and were serving the 22 HIV care and treatment clinics.

### TB screening

At enrolment and each follow-up visit (i.e., at 2 weeks, then monthly for the first 3 months, and then quarterly for the remaining follow-up period), adults aged > 12 years and children aged 0–12 years were screened for TB symptoms. Per protocol, adults were screened for four TB symptoms (cough, fever, night sweats, and weight loss) of any duration. Children were screened for weight loss or failure to thrive (no weight gain over 3 months), enlarged lymph nodes (more than 1 × 1 cm), cough for ≥2 weeks, fever for ≥2 weeks, fatigue/reduced playfulness for ≥2 weeks, and profuse night sweats for ≥2 weeks [15].

### Sputum collection

Patients who screened positive for at least one TB symptom were requested to provide four sputa samples: two were provided on the screening day (spot 1 and 2) and two on the following day. On day 2, one sputum sample was collected at home early in the morning (morning sample), and another sample was taken at the clinic (spot 3). Patients in the smear arm were enrolled in XPRES before Xpert MTB/RIF instrument implementation; therefore, spots 1 and 3 were tested only with Ziehl–Nielson smear at the peripheral laboratory. However, if patients in the smear arm screened positive for TB during a follow-up appointment after Xpert MTB/RIF instrument implementation, spots 1 and 3 were tested by Xpert MTB/RIF at the peripheral laboratory or point of care sites. For the Xpert arm, all spot 1 and 3 samples were tested by Xpert MTB/RIF either at the peripheral laboratory or at point of care sites.

Spot 2 and morning samples were submitted to the National TB Reference Laboratory for liquid culture, Mycobacteria Growth Indicator Tube (MGIT 960 instrument, Becton Dickinson, Sparks, MD, USA) and drug susceptibility testing.

### TB treatment outcome and definitions of terms

TB treatment outcomes were defined as unfavorable (death, treatment failure, or loss-to-follow-up) or favorable (treatment completion, cure). TB was microbiologically confirmed from a biological specimen that tested positive via smear or Xpert MTB/RIF. Presumptive TB refers to a patient who presented with symptoms suggestive of TB [16]. Time-to-treatment was defined as time from sputum collection to TB treatment initiation. Empiric treatment was defined as initiating treatment without or before receiving a TB-positive test result [12]. Following TB treatment initation, a follow-up smear test was performed per the national tuberculosis program guidelines, i.e. at the end of month 2, 3, month 5 or 6. A month 5 or 6 smear was used to determine if a patinets was cured or failed treatment. A month 5 or 6 smear was used to determine if a patient was cured or failed treatment [6].

### Data collection

Data were prospectively collected using standardized case report forms between August 2012 and November 2014 and were double-entered into a Clindex database (Fortress Medical Systems, Minneapolis, MN, USA). Inconsistencies were identified through logic checks and were checked against the original case report form. Where possible, inconsistencies and missing data were corrected through review of patient charts.

### Statistical analysis

Data were analyzed using STATA (Release 14, StataCorp, College Station, TX USA) [17]. We used univariate and multiple logistic regression models to describe and compare the demographic and clinical characteristics of patients. To examine TB incidence hazard ratio, we used Cox proportional hazards models among patients with TB diagnosed via smear or the Xpert MTB/RIF algorithm. All analyses were adjusted for within-facility correlation. *P* values < 0.05 were considered statistically significant. The trial adhered to CONSORT guidelines and the profile will be summarised using a CONSORT flow chart [18].

## Results

**Fig. 1:**
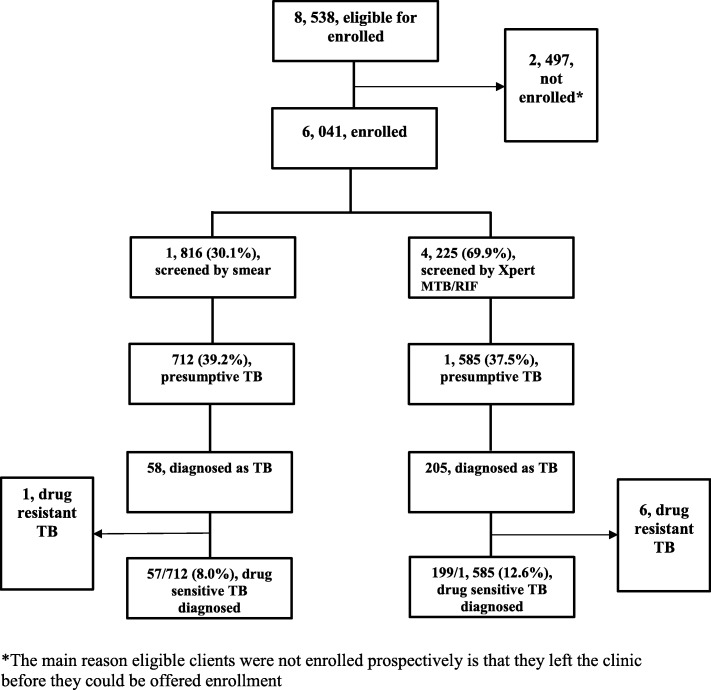
Enrollment*, screening by smear or by Xpert MTB/RIF, and TB diagnosis in Botswana

Among 6041 participants, 1816 were enrolled and screened using sputum-smear microscopy (smear arm), and 4225 were screened using Xpert MTB/RIF (Xpert arm) from August 2012 and November 2014. After the initial consultation and follow up, 2297 (smear arm, 712; Xpert arm, 1585) were designated as presumptive TB patients. Follow-up of last patient was completed by the end of June 2015. A total of 256 (199/4225, 4.7% and 57/1816, 3.1%, in Xpert and smear arm, respectively, odds ratio [OR], 1.53, *P = 0.005* or with follow-up data 199 per 2985 and 57 per 1582 person-years of follow-up in Xpert and smear arms, respectively; adjusted incidence rate ratio, 9.07; 95% confidence interval [CI]: 4.70–17.48; *p* < 0.001) were diagnosed with and treated for drug-sensitive TB (Fig. 1). The rates of TB among presumptive TB patients were 12.6% (199/1585) in the Xpert arm and 8.0% (57/712) in the smear arm (OR, 1.65; 95% CI: 1.21–2.24; *p* = 0.001; Fig. 1).

Table 1 shows demographic characteristics between the smear arm (1816) and the Xpert arm (4225), and Table 2 shows characteristics of patients with or without TB. There were no statistically significant differences among characteristics in both Table 1 and Table 3. Patients with TB (256; Table 2) were more likely to have a CD4 count < 200 cells/mm^3^ (adjusted OR, 2.16; 95% CI: 1.72–2.72; *p* < 0.001) and a body mass index < 18.5 (aOR, 2.41; 95% CI: 1.80–3.23; *p* < 0.001) and were less likely to be younger than 35 years or use alcohol (aOR, 0.68; 95% CI: 0.46–0.99; *p* = 0.047) than participants without TB (5785; Table 2). Sex, previous history of TB treatment, current or history of smoking, and occupation as a miner were similar between participants, with or without TB.

**Table 1:** Characteristics of HIV-patients screened for tuberculosis via smear or Xpert MTB/RIF in Botswana

	Xpert arm		Smear arm				
Characteristic	N	n (%)	N	n (%)	aOR	95% CI^b^	*p*-value
Age^a^ < 35 years	4224	2333 (55.2)	1816	961 (52.9)	1.11	0.98–1.26	0.083
Gender, female	4225	2796 (66.2)	1816	1234 (68.0)	0.87	0.73–1.05	0.134
CD4 count < 200 cells/mm^3^	4124	1658 (40.2)	1797	662 (36.8)	1.15	0.97–1.37	0.093
BMI < 18.5	4225	827 (19.6)	1816	393 (21.6)	0.85	0.69–1.05	0.127
Previous TB	4219	452 (10.7)	1814	194 (10.7)	1.03	0.77–1.39	0.827
Smoking^b^	4216	823 (19.5)	1809	339 (18.7)	1.03	0.77–1.38	0.840
Alcohol use	4217	918 (21.8)	1809	403 (22.3)	0.95	0.73–1.22	0.660
Mine	4217	209 (5.0)	1809	94 (5.2)	0.89	0.63–1.27	0.514

Abbreviations: *aOR* adjusted odds ratio, *CI* confidence interval, *BMI* body mass index

^a^ The majority of HIV-positive children were seen at a centralized center, as a result < 0.5% (30/6041) of children were enrolled at study sites. ^b^Current or ex-smoker

**Table 2:** Characteristics of HIV-patients with and without tuberculosis screened in HIV clinics in Botswana

	Patients with TB		Patients without TB				
Characteristic	N	n (%)	N	n (%)	aOR	95% CI	*p-*value
Age < 35 years^a^	256	108 (42.2)	5784	3186 (55.1)	0.73	0.55–0.97	0.034
Gender, female	256	128 (50.0)	5785	3902 (67.5)	0.76	0.52–1.12	0.155
CD4 count < 200 cells/mm^3^	252	158 (62.7)	5669	2162 (38.1)	2.16	1.72–2.72	< 0.001
BMI < 18.5	256	104 (40.6)	5785	1116 (19.3)	2.41	1.80–3.23	< 0.001
Previous TB	255	33 (12.9)	5778	613 (10.6)	0.84	0.56–1.27	0.404
Smoking^b^	256	74 (28.9)	5769	1088 (18.9)	1.35	0.96–1.89	0.081
Alcohol use	256	55 (21.5)	5770	1266 (21.9)	0.68	0.46–0.99	0.047
Miner	256	25 (9.8)	5770	278 (4.8)	1.58	0.90–2.79	0.105

Abbreviations: *aOR* adjusted odds ratio, *CI* confidence interval, *BMI* body mass index

^a^ The majority of HIV-positive children were seen at a centralized center, as a result < 0.5% (30/6041) of children were enrolled at study sites. ^b^Current or ex-smoker

**Table 3:** Characteristics of HIV-patients with tuberculosis screened using smear or Xpert MTB/RIF in Botswana

	TB patients screened using smear		TB patients Screened using Xpert MTB/RIF				
Characteristic	N	n (%)	N	n (%)	aOR	95% CI	*p*-value
Age < 35 years^a^	57	25 (43.9)	199	83 (41.7)	1.09	0.60–1.97	0.773
Gender, female	56	29 (51.8)	199	99 (49.7)	1.04	0.36–2.98	0.946
CD4 count < 200 cells/mm^3^	57	39 (68.4)	196	119 (60.7)	1.36	0.65–2.83	0.391
BMI < 18.5	57	31 (54.4)	199	75 (37.7)	1.73	0.83–3.61	0.137
Previous TB	57	7 (12.3)	198	26 (13.1)	0.68	0.23–2.01	0.471
Smoking^b^	57	16 (28.1)	199	58 (29.1)	0.78	0.36–1.70	0.516
Alcohol use	57	13 (22.8)	199	42 (21.1)	1.31	0.55–3.12	0.522
Miner	57	4 (7.0)	199	21 (10.6)	1.01	0.21–4.77	0.987
TB symptoms							
Cough	57	41 (71.9)	199	138 (69.3)	0.96	0.37–2.51	0.928
Fever	57	27 (47.4)	199	79 (39.7)	1.72	1.13–2.62	0.014
Night sweats	57	19 (33.3)	199	79 (39.7)	0.57	0.27–1.20	0.133
Weight-loss	57	41 (71.9)	199	121 (60.8)	1.43	0.65–3.11	0.354

Abbreviations: *aOR* adjusted odds ratio, *CI* confidence interval

^a^ The majority of HIV-positive children were seen at a centralized center, as a result < 0.5% (30/6041) of children were enrolled at study sites. ^b^ current or ex-smoker

Table 3 shows demographic characteristics among TB patients in the smear and Xpert arms. For the majority of the characteristics there were no statistically significant difference in both arms; however, TB patients in the smear arm were more likely to have fever symptom (aOR, 1.72; 95% CI: 1.13–2.62; *p* = 0.014) than TB patients in the Xpert arm (Table 3).

### TB treatment outcomes

As of November 2014, TB treatment outcomes were available for 203/256 (79.3%) patients (smear arm, 46; Xpert arm, 157). The other 53 TB patients were transferred out or not evaluated. Although not statistically significant, patients with TB diagnosed via smear were more likely to have unfavorable treatment outcomes than those with TB diagnosed via Xpert MTB/RIF (adjusted hazard ratio: 1.40; 95% CI: 0.75–2.26; *p* = 0.268; Table 4).

**Table 4:** Tuberculosis treatment outcomes among PLHIV screened using smear or Xpert MRB/RIF in Botswana

Treatment outcomes	TB patients screened by smear	TB patients screened Xpert MTB/RIF	Adjusted Hazard ratio^a^	95% CI^a^
	*n* = 57	*n* = 199		
Unfavorable outcome^b^	10 (21.7%)	21 (13.4%)	1.40	0.75–2.26
Favorable outcome^c^	36 (78.3%)	136 (86.6%)		
Subtotal	46	157		
Transferred out or not evaluated	11	42		

^a^adjusted for inter-facility difference, *P value = 0.268*

^b^death, lost-tofollow-up, treatment failure

^c^cure, completed treatment

### Factors affecting treatment outcomes

#### Placement of Xpert MTB/RIF at a point of care versus a laboratory

Among 157 patients with TB diagnosed using Xpert MTB/RIF and with treatment outcome data, 48 and 109 received their diagnosis from point of care and laboratory sites, respectively. A non-significant higher unfavorable treatment outcome was observed among TB patients screened and diagnosed at a point of care, compared to those screened and diagnosed at a laboratory: 7/48 (14.6%) at point of care sites vs. 14/109 (12.8%) at laboratory sites (aOR, 1.16; 95% CI: 0.37–3.66; *p* = 793).

### Time-to-treatment

Median days from sputum collection to TB treatment among patients in the smear and in the Xpert arms were 22 days (interquartile range [IQR], 3–51) and 6 days (IQR, 2–17), respectively (*p* = 0.005, Mann-Whitney test; Table 5). Time-to-treatment in the Xpert arm was significantly shorter, and unfavorable outcome, however, was similar among smear arm and Xpert arms, 4/35 (11.4%) unfavorable outcomes vs 10/121 (8.3%) in the Xpert arm (aOR, 1.43; 95% CI: 0.63–3.27; *p* = 0.374)*.*

**Table 5:** Time-to-treatment between patients screened for tuberculosis using smear or Xpert MRB/RIF in Botswana

	Smear arm	Xpert MTB/RIF arm	*p*-value^a^
	*n* = 42	*n* = 159	
Median days to initiation of TB treatment^b^, IQR	22 (3–51)	6 (2–17)	0.005

^a^ Mann-Whitney test

^b^ Median days – days from specimen collection date to anti-tuberculosis treatment initiation

### Empiric treatment

A total of 53% (136/256) TB patients were initiated empirically on anti-TB treatment without or before receiving TB-positive test results (smear, Xpert or culture). Among the 136 treated empirically, 34 were later reported as MTB culture-positive (13 in smear and 21 in Xpert arm). These culture-positive report, however, were presented to a treating clinician after patients were already initiated (empirically) on anti-TB treatment. In the smear arm, the empiric TB treatment rate (39/57 [68.4%]) was significantly higher than in the Xpert arm (97/199 [48.7%]; aOR, 2.28; 95% CI: 1.24–4.20; *p* = 0.011).

### Microbiologically confirmed TB

Patients in the Xpert arm who submitted at least one sputum sample for testing (102/173 [59.0%]) were more likely to be diagnosed with microbiologically confirmed TB than those patients in the smear arm (18/43 [41.9%]; aOR, 2.00; 95% CI: 1.01–3.96; *p* = 0.048).

### Loss-to-follow-up

The rate of loss-to-follow-up among the two, smear and Xpert arms, was similar with 2.2% (1/46) vs. 2.5% (4/157), aOR, 1.18, 95% CI: 0.09–14.76, *p = 0.900*), respectively.

## Discussion

Incorporating Xpert MTB/RIF into national TB policy and into the TB diagnostics algorithm was a relatively smooth process in Botswana. Implementing Xpert MTB/RIF together with the pragmatic clustered randomized trial under routine program conditions allowed us to evaluate the impact of Xpert MTB/RIF on unfavorable treatment outcomes by comparing data from patients diagnosed with TB using Xpert MTB/RIF or sputum-smear microscopy algorithms. In our settings, Xpert MTB/RIF demonstrated superiority to smear in microbiologically confirming TB, reducing time-to-treatment, and reducing empiric treatment. The effect of Xpert MTB/RIF on reducing unfavorable treatment outcome was higher than conventional smear on patient-level tuberculosis treatment though the difference has not reached statistically significant level In our setting, GeneXpert instrument or environmental related factors such as power supply and temperature that potentially affect Xpert MTB/RIF performance were controlled [19].

It is not clear why reduced time-to-treatment initiation and reduced empiric treatment did not translate to improved patient-level treatment outcomes in our study. All patients in our study were HIV-positive, ART naïve at the time of enrolment with similar patient characteristics, including CD4 cell count, suggesting that immunologic status and/or clinical presentation did not affect TB treatment outcomes among comparison arms (smear versus Xpert).

This trial was one of the four clustered randomized trials, including those in South Africa and Brazil, that investigated the effect of Xpert MTB/RIF on TB treatment outcomes compared to smear [8, 10, 11]. Although Xpert MTB/RIF has higher sensitivity and shorter turnaround time than smear [7, 9, 10], none of the four trials, including the present study, found improved TB treatment outcomes in the Xpert arm [8, 10, 11], suggesting that replacing smear with Xpert MTB/RIF is not sufficient to drive clinical outcome improvement. Furthermore, Theron et al. and Yoon et al. reported 2-month mortality rates [20, 21], and Cox et al., Calligaro et al., and Churchyard et al. reported 3-month and/or 6-month mortality rates comparing smear with Xpert MTB/RIF [10, 22, 23]. None of these recent studies showed mortality benefits in patients with TB diagnosed via Xpert MTB/RIF compared with those with TB diagnosed via smear. However, Auld et al. observed a 12-month mortality benefit for TB patients receiving enhanced care (defined as intensified case finding with additional staff support to actively trace patients who missed clinic appointments) compared with those who received standard of care [24].

Given the available evidence, it is becoming clear that TB control would require more than improvements in the sputum sample processing speed and accuracy of molecular diagnostics, although TB diagnostics help identify more patients with TB. Stop TB Partnership and WHO emphasize that evaluating new TB diagnostics is an essential component after introducing a new diagnostics tool into a national TB program activities. Ideally, new molecular diagnostics would lead to an epidemiological impact, such as reduced TB disease transmission at a population level, and improved patient-level outcomes [25]. Over 145 countries have implemented the Xpert MTB/RIF assay as of December 2016 [26]. However, Xpert has not improved patient-level outcomes, which suggests that Xpert is not likely to have a population-level impact.

Our findings suggest that the Botswana national TB program and similar settings could consider other types of strategic innovations (e.g., prevention, diagnostic, and therapeutic) beyond reliance on technology. Auld et al’s emphasis on intensified case finding and active tracing of TB patients to improve retention in care is an example of such strategies mentioned above. TB preventive therapy is another effective strategy; however, global uptake remains low. As of 2016, only 13% of eligible children younger than 5 years and 38% of PLHIV newly enrolled in care were receiving TB preventive therapy worldwide [27]. Scale-up of TB preventive therapy is essential, especially as Badje et al. reported that TB preventive therapy reduced mortality rates by 37%, independent of ART status [28]. WHO now recommends establishing or scaling up targeted TB preventive therapy for HIV-negative household contacts of people with bacteriologically confirmed TB in TB endemic countries because this group is at higher risk of TB than the general population [29]. Furthermore, given that Xpert MTB/RIF can rapidly diagnose drug-resistant TB, extra-pulmonary TB, and pediatric TB and that the Xpert Ultra assay requires less infrastructure, further research is needed to explore pre- and post-diagnostic test-related factors in the TB diagnostic cascade in health systems [30]. In addition, systematic reviews and meta-analyses summarizing potential barriers to successful treatment outcomes of patients with TB diagnosed via Xpert MTB/RIF or smear are essential.

Our study has several limitations. First, in both the Xpert MTB/RIF and smear arms, close to 20% of patients were either transferred out or not evaluated. Second, our analysis is a sub-study focusing on comparing treatment outcomes among patients with TB diagnosed via smear or Xpert MTB/RIF. Detailed results on culture and drug susceptibility tests were not included in this report because these findings will be published with the main XPRES study. Third, we were unable to control pre- and post-diagnostic test-related factors in the TB diagnostic cascade, such as delayed sputum sample submission, delayed sample transportation, lack of regular maintenance of GeneXpert instruments, and whether laboratories used the most recent version of the Xpert cartridge (G3 vs. G4). Fourth, for some TB patients diagnosis was during the follow-up period and the effect of follow-up visit on repeat TB screening, quality of sample collection and on the diagnosis of TB was not measured or adjusted in to the analysis. Lastly, we attempted to adjust for within facility difference when Wilcoxon rank-sum test analysis was conducted. However, STATA does not have a syntax for such adjustment when Wilcoxon rank-sum test analysis ran. Thus, Wilcoxon rank-sum test analysis was not adjusted for clustering within clinics.

## Conclusions

Our findings indicate a non-significant higher unfavorable treatment outcomes among TB patients diagnosed using conventional smear, compared to TB diagnosis informed by Xpert. However, compared to the smear arm, more patients in the Xpert arm received a TB diagnosis, had a microbiologically confirmed TB, and had a shorter time-to-treatment, and had a lower empiric treatment rate. Further research is needed to identify potential gaps in the Botswana health system and similar settings.

## Data Availability

The authors confirm that, for approved reasons, some access restrictions apply to the data underlying the findings. Although the patient-level data do not include patient names, this IRB decision is in the interest of ensuring patient confidentiality. An individual may email the lead author (tagizew@cdc.gov) or the CDC division of Global HIV/AIDS science office (gapmts@cdc.gov) to request the data.

## Abbreviations

AOR: Adjusted Odds Ratio
ART: Antiretroviral Therapy (ART)
BMI: Body Mass Index
CI: Confidence Interval
CONSORT: Consolidated Standards of Reporting *Trials*
CRT: Cluster Randomised Trial
IQR: Interquartile Range
MIGT: Mycobacteria Growth Indicator Tube
PLHIV: People living with HIV
STATA: South Texas Art Therapy Association
WHO: World Health Organization
XPRES: Xpert Package Rollout Evaluation Study
